# Financial Resources and Later-Year Relocation From the Annuitized Assessment of Income and Assets

**DOI:** 10.1093/geroni/igaa057.162

**Published:** 2020-12-16

**Authors:** Jun-Hong Chen, Sojung Park

**Affiliations:** 1 Washington University in St Louis, Saint Louis, Missouri, United States; 2 Washington University in St. Louis, St. Louis, Missouri, United States

## Abstract

Solid evidence has shown financial resources play important roles in housing decisions among older adults. Despite the growing research on the joint assessment of income and assets as valid economic well-being, little attention is paid to its role in relocation in old age. Drawing from the Behavioral Model of Elderly Migration, this study examined to what extent financial resources are associated with the likelihood of moving in later years. The data came from the 2017 Panel Study of Income Dynamic (PSID). A sample of 1354 people, 65 years and older, was used in the analyses. We used the annuitized approach, which is different from conventional approaches that assume people draw down all available assets to satisfy daily needs and leave no assets for use in later years. We (1) assessed annuitized assets based on the 2019 IRS Mortality Table, (2) assessed yearly income using supplementary income (i.e. income plus non-discretionary expense). A final indicator of the summed score was used in a logistic regression to predict the likelihood of moving. A set of covariates known to affect later- year relocation at an individual level (e.g. health condition, living arrangement change), environmental level (e.g. rural, non-metro area) are controlled for. In clear conflict with previous studies, we found annual financial resources did not significantly influence relocation among older adults. The notable absence of the well-known role of the economic factor provides critical initial evidence about the importance of simultaneous assessment of financial resources for the literature on later year relocation.

